# Individualized Behavioral Management Techniques to Facilitate and Maintain Dental Treatment in a Child With Autism Spectrum Disorder: A Case Report

**DOI:** 10.7759/cureus.109519

**Published:** 2026-05-23

**Authors:** Liliet Dominguez Gonzalez, Richard M Cavero, Mayree Ferreira-Alfonzo, Giusel Almaguer, Noelia Ramirez Garcia

**Affiliations:** 1 Dentistry, Family Cosmetic Dentistry, Miami, USA; 2 General Practice, Metro Dental Associates, Denville, USA; 3 Dentistry, Private Practice, Miami, USA; 4 Dentistry, Melone Orthodontic Associates, Tenafly, USA; 5 Dentistry, Smile Express, Bloomfield, USA

**Keywords:** autism spectrum disorder, behavioral management, caregiver involvement, dental anxiety, desensitization, non-pharmacological management, pediatric dentistry, sensory sensitivity, special needs dentistry, tell-show-do

## Abstract

Autism spectrum disorder (ASD) presents significant challenges in dental practice due to impairments in communication, sensory processing, and behavioral regulation. This case report describes the management of a five-year-old male child with ASD who exhibited anxiety, hypersensitivity to auditory stimuli, and limited cooperation during dental visits. An individualized, non-pharmacological behavioral approach was implemented, including desensitization, the tell-show-do technique, non-verbal communication, caregiver involvement, and environmental modifications aimed at reducing sensory overload. Treatment was conducted over multiple visits, initially focusing on behavioral adaptation before progressing to preventive and restorative procedures. The child demonstrated progressive improvement in tolerance and cooperation, allowing initiation and maintenance of intraoral dental treatment without the use of sedation or general anesthesia. This case highlights the effectiveness of individualized behavioral management techniques in facilitating and sustaining cooperation and improving clinical outcomes. It also emphasizes the importance of a child-centered approach, consistency in clinician behavior, and caregiver support in managing children with ASD in the dental setting.

## Introduction

Autism spectrum disorder (ASD) is a neurodevelopmental condition characterized by persistent deficits in social communication and the presence of restricted or repetitive patterns of behavior [1]. These features can significantly impact the delivery of dental care, particularly in children, where cooperation and communication are essential for successful treatment outcomes [2].

Children with ASD frequently present with sensory sensitivities, including heightened responses to auditory, visual, and tactile stimuli [3]. The dental environment, with its unfamiliar sounds, bright lights, and physical proximity, can therefore act as a source of significant stress and anxiety. In addition, communication challenges and difficulty adapting to new environments may further complicate routine dental procedures and limit access to care [4]. Children with ASD also demonstrate higher rates of dental caries and treatment avoidance, increasing the need for effective behavioral strategies to improve both immediate cooperation and long-term oral health outcomes. Children with ASD are at increased risk for poor oral health, including higher rates of dental caries and gingival inflammation, often due to difficulties maintaining adequate oral hygiene and reduced tolerance for professional dental interventions [5].

To address these challenges, dental professionals must adopt individualized, child-centered approaches that prioritize behavioral management and gradual adaptation to the clinical environment. Non-pharmacological strategies, such as desensitization, structured communication techniques, caregiver involvement, and the tell-show-do method, have been shown to improve cooperation and reduce the need for pharmacological interventions [6,7]. Recent evidence has also demonstrated the effectiveness of behavioral guidance techniques, sensory-adapted dental environments, and structured dental adaptation strategies in improving cooperation and facilitating dental treatment in children with ASD [8-10].

Additional studies have emphasized the importance of clinician preparedness, individualized management approaches, and behavioral guidance in improving treatment acceptance and reducing anxiety in children with ASD [11,12]. This report describes the clinical presentation and management of a child with ASD, highlighting the effectiveness of a structured and individualized behavioral approach in facilitating successful dental treatment.

## Case presentation

A five-year-old male child diagnosed with ASD was referred to the dental clinic for evaluation and treatment. The caregiver reported previous unsuccessful dental visits due to severe anxiety, refusal to sit in the dental chair, and episodes of agitation.

Upon presentation, the child demonstrated limited verbal communication, poor eye contact, repetitive hand movements, and hypersensitivity to auditory stimuli. Initial attempts at clinical examination were met with resistance, including head turning, vocal distress, and attempts to leave the operatory.

A limited clinical examination revealed generalized plaque accumulation and multiple carious lesions affecting primary molars. Radiographic evaluation was not feasible during the initial visit because of limited cooperation.

**Figure 1:**
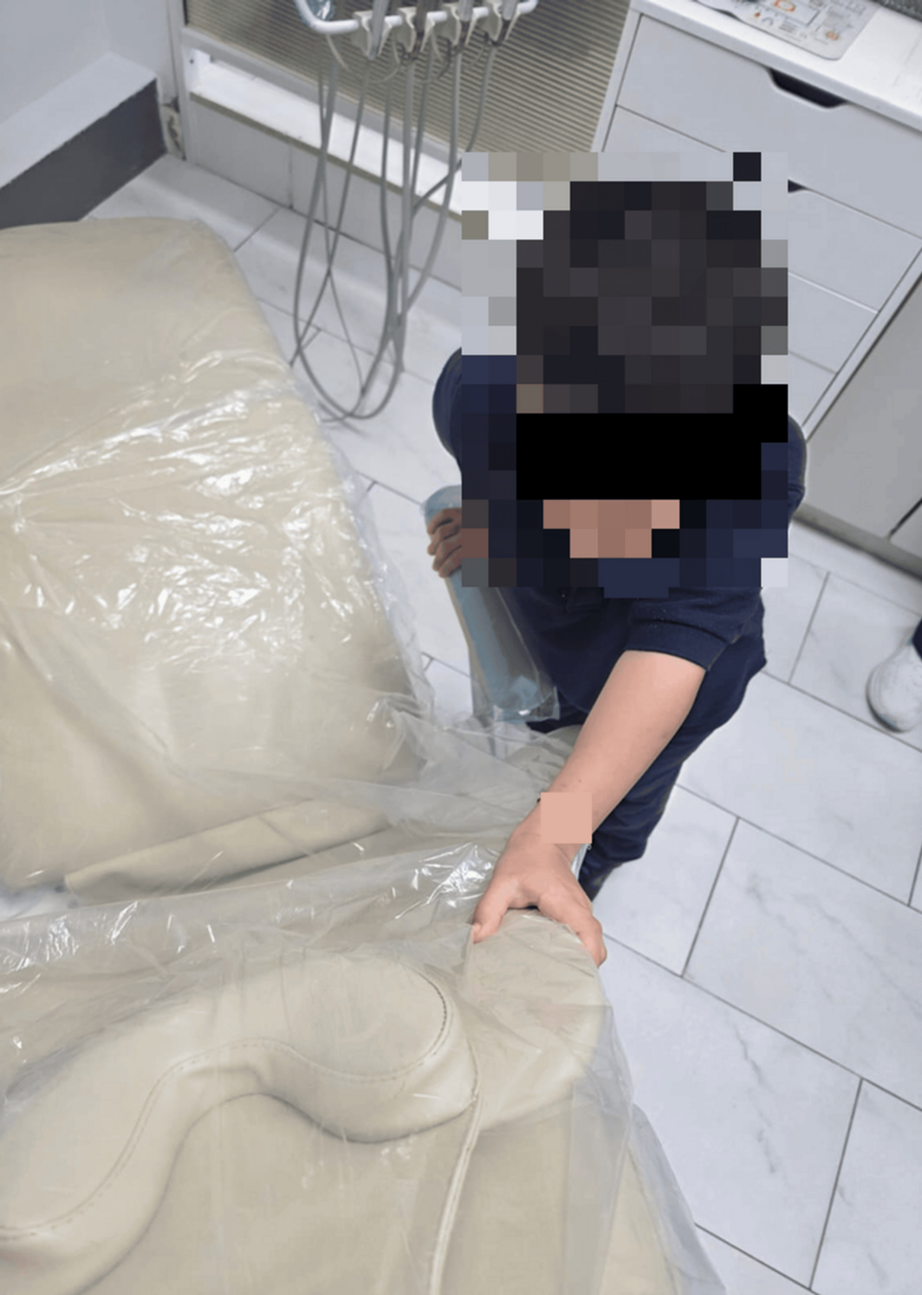
Behavioral acclimatization of a pediatric patient with autism spectrum disorder in the dental operatory Initial desensitization phase in which the child is encouraged to interact with the dental chair and the surrounding environment to promote familiarity, adaptation to the dental environment, and reduction of anxiety. This non-pharmacological behavioral approach helps facilitate cooperation prior to clinical treatment. Patient identity has been protected to ensure privacy.

A phased treatment approach was developed, prioritizing behavioral adaptation before initiating definitive dental treatment. A structured, non-pharmacological behavioral management protocol was implemented. As shown in Figure 1, the child was gradually introduced to the dental environment through short, non-invasive visits aimed at desensitization and behavioral adaptation.

**Figure 2:**
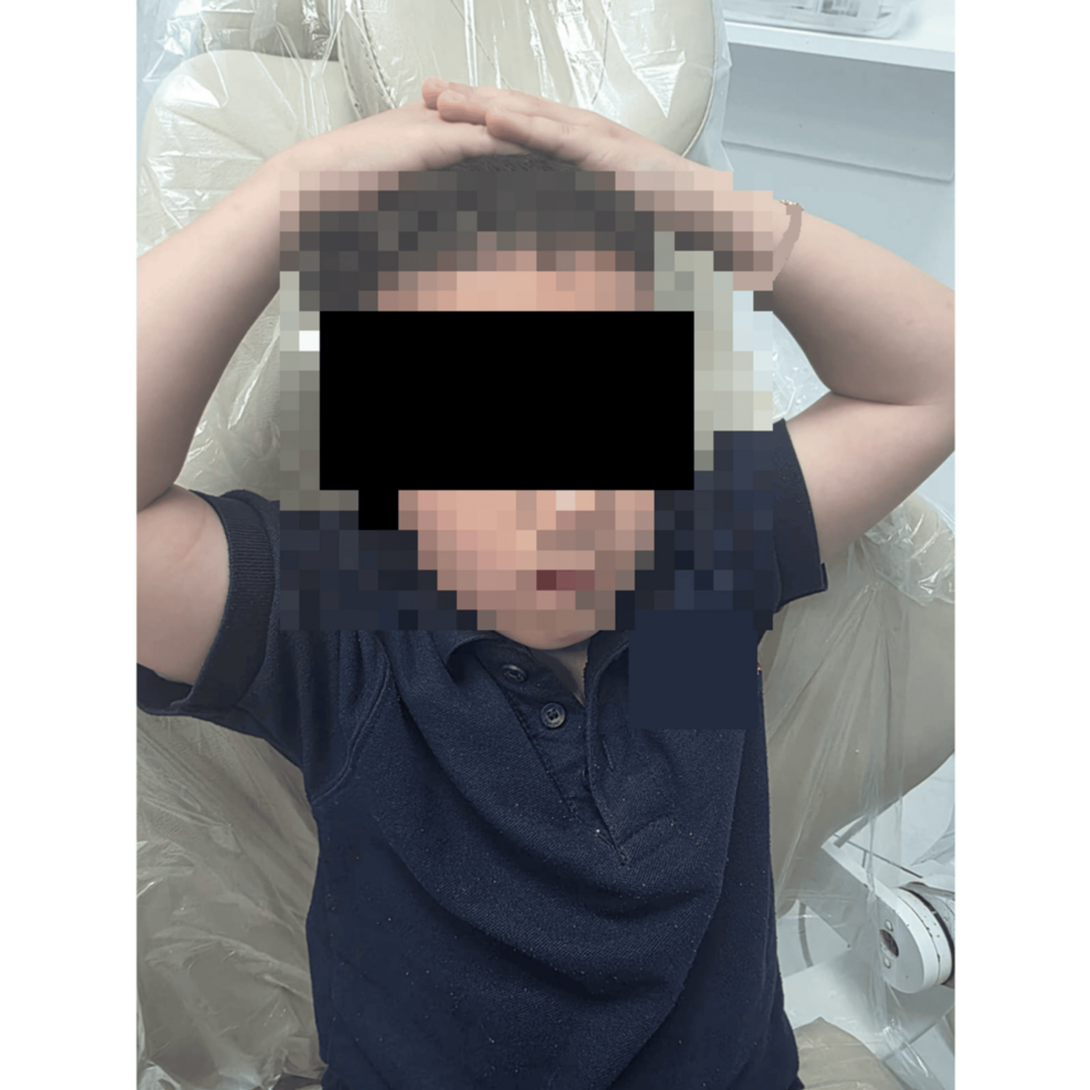
Mild behavioral distress observed during dental chair positioning in a child with autism spectrum disorder The child demonstrated mild stress and self-regulatory behaviors while seated in the dental chair. Behavioral management techniques, including reassurance, voice modulation, and gradual desensitization, were employed to reduce anxiety and facilitate cooperation. Patient identity has been protected to ensure privacy.

During initial seating in the dental chair, the child demonstrated mild behavioral distress and self-regulatory responses. Figure 2 demonstrates the use of reassurance and gradual behavioral guidance techniques to reduce anxiety and facilitate adaptation to the clinical environment.

**Figure 3:**
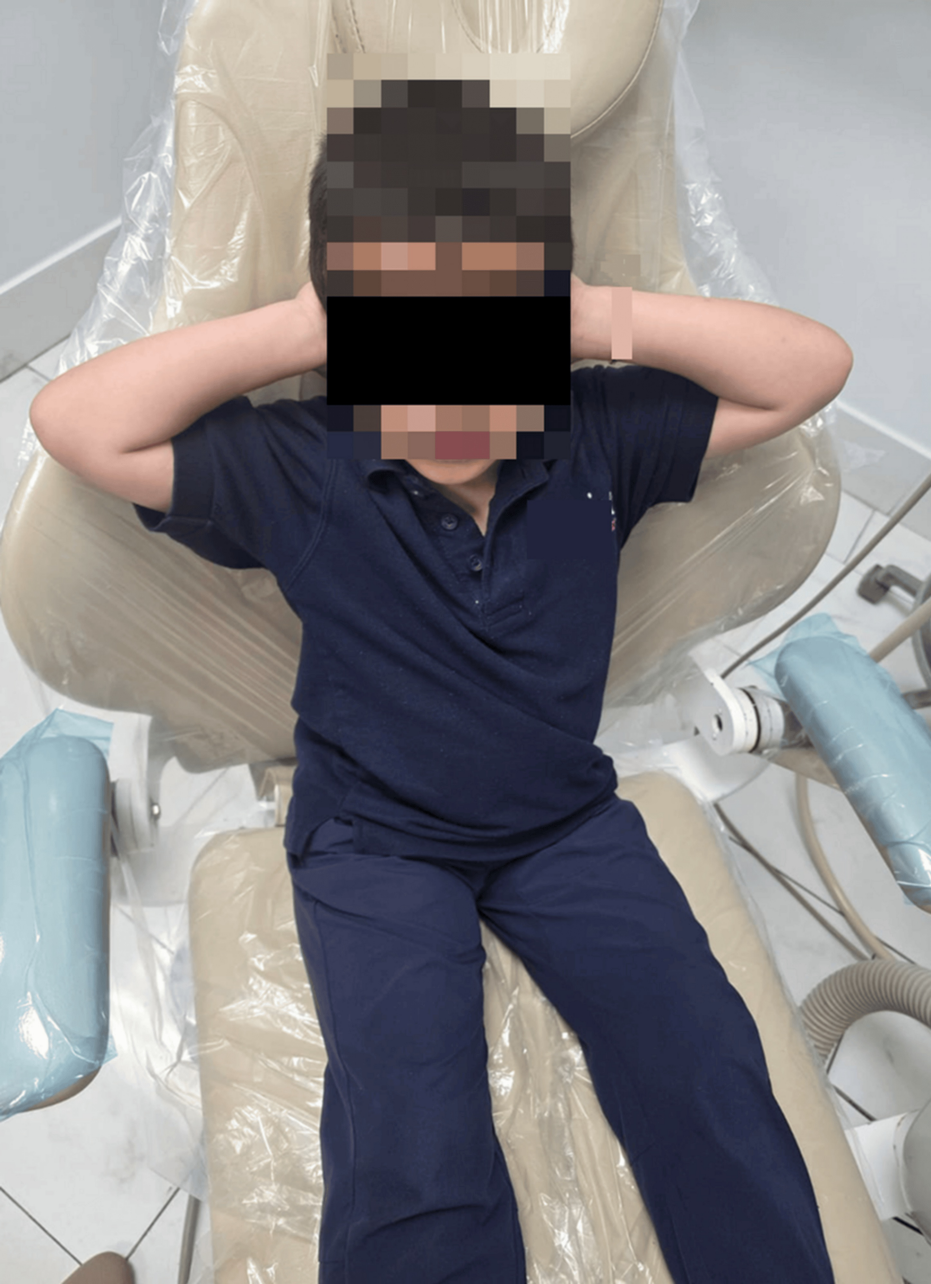
Auditory sensitivity and protective response to dental operatory stimuli in a child with autism spectrum disorder The child demonstrated a protective response by covering the ears, indicating sensitivity to auditory stimuli within the dental environment. Noise from dental instruments, particularly high-speed handpieces, may act as a trigger for stress in children with autism spectrum disorder. Behavioral management strategies include minimizing noise exposure, using low-noise equipment, and gradual sensory adaptation. Patient identity has been protected to ensure privacy.

Environmental modifications were introduced, including the reduction of noise levels and minimization of exposure to dental instruments to decrease sensory overload. Figure 3 highlights the patient’s auditory hypersensitivity during exposure to operatory stimuli within the dental environment.

**Figure 4:**
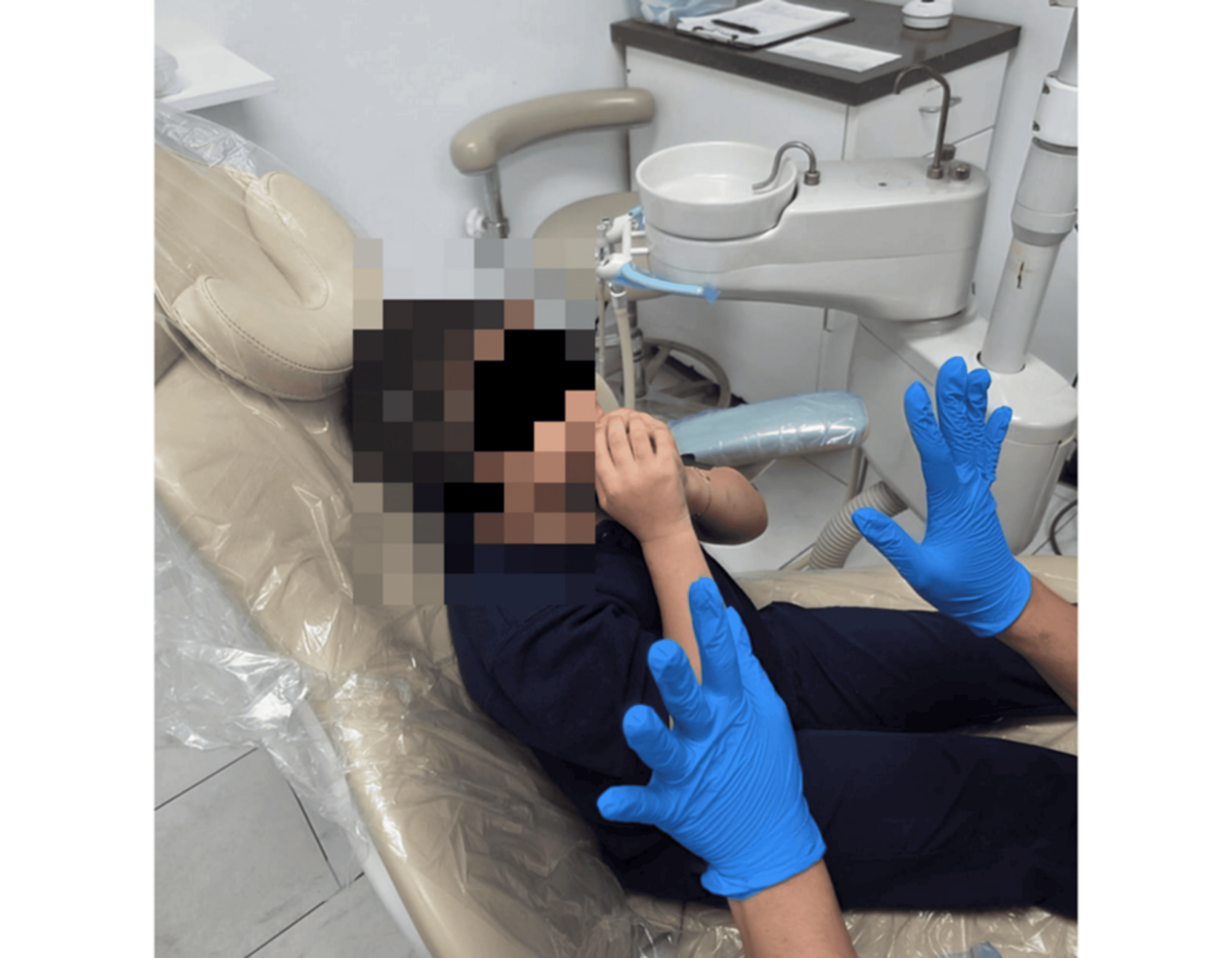
Use of non-verbal communication and rapport-building techniques in a child with autism spectrum disorder The clinician engages the child using non-verbal communication, including hand gestures and a calm, friendly approach, to establish trust and reduce anxiety. Play-based interaction and positive reinforcement were used to promote cooperation and create a supportive clinical environment. Patient identity has been protected to ensure privacy.

Non-verbal communication and rapport-building techniques were used to establish trust and promote patient comfort within the dental environment. Figure 4 illustrates the use of non-verbal interaction and behavioral guidance to facilitate cooperation and reduce anxiety during the clinical encounter.

**Figure 5:**
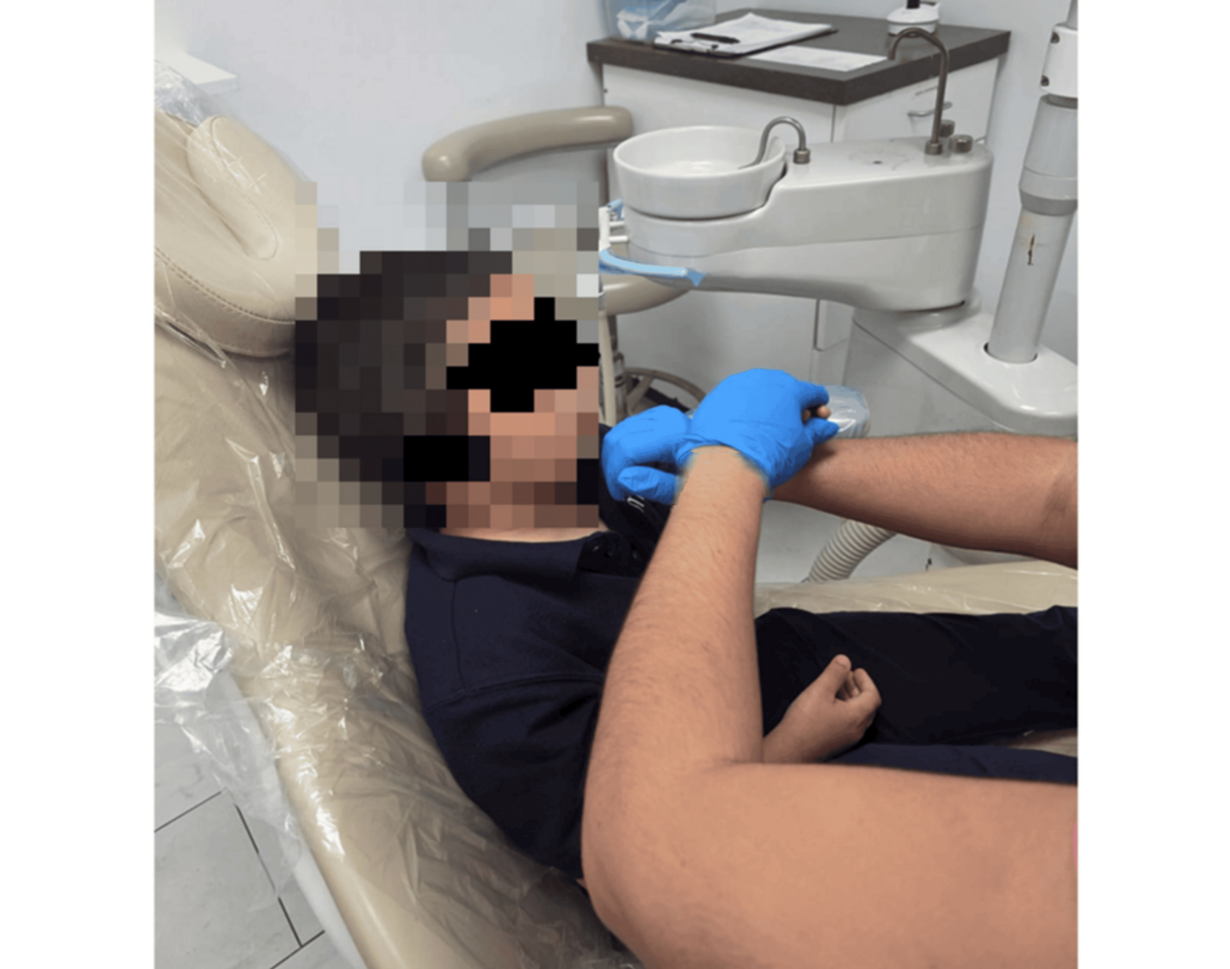
Tactile and non-verbal communication techniques used to promote reassurance in a child with autism spectrum disorder The clinician used gentle tactile interaction and non-verbal communication to establish a sense of safety and trust. Hand guidance and play-based engagement were used to reduce anxiety and facilitate cooperation. These techniques are particularly beneficial for children with autism spectrum disorder, who may respond more positively to non-verbal cues. Patient identity has been protected to ensure privacy.

Tactile guidance and non-verbal reassurance techniques were implemented to enhance the child’s sense of safety and cooperation during clinical interaction. Figure 5 demonstrates the use of tactile and behavioral communication strategies to promote reassurance and patient comfort during dental care.

**Figure 6:**
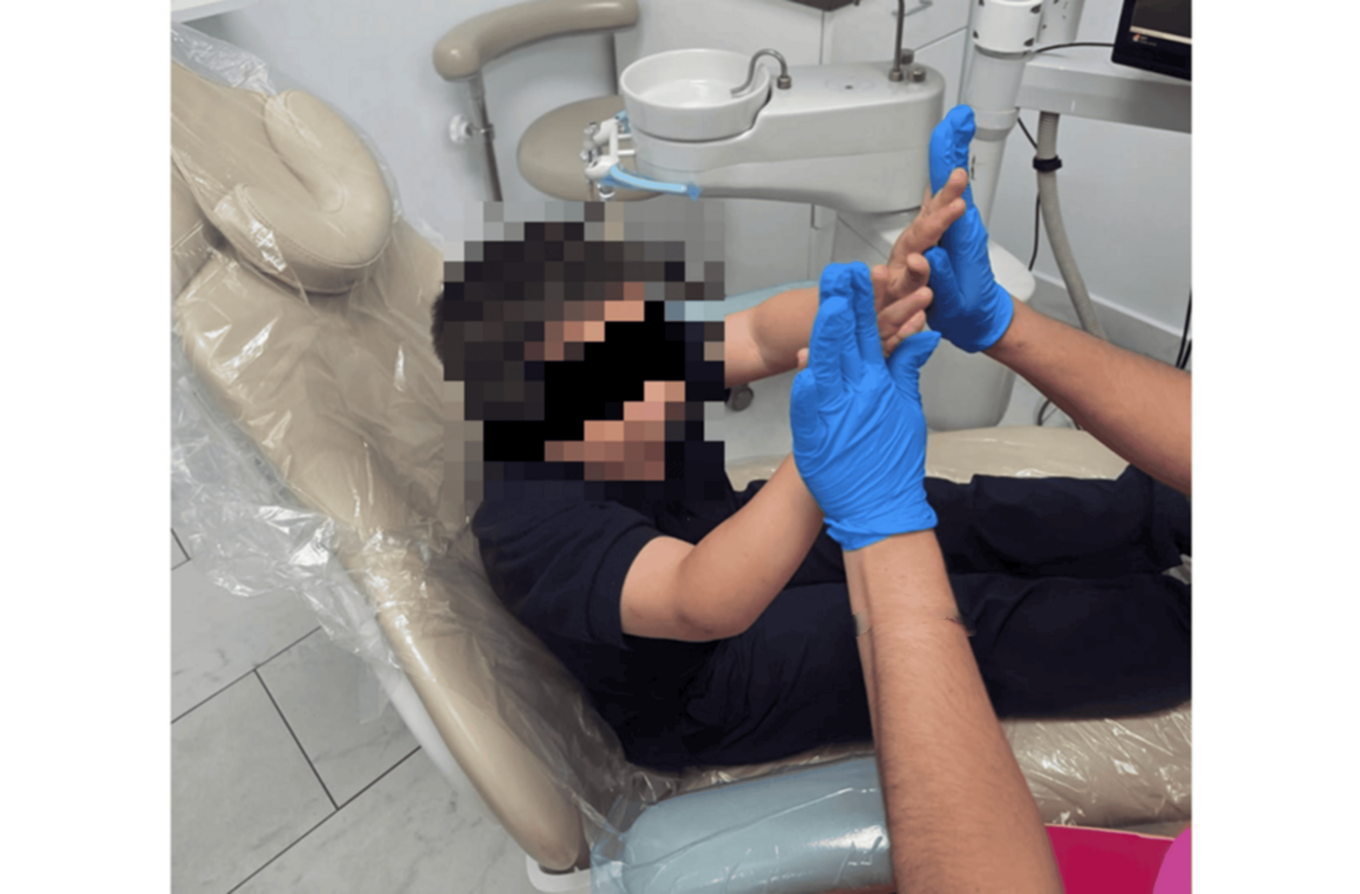
Successful behavioral adaptation using the tell-show-do technique in a child with autism spectrum disorder The child demonstrated improved cooperation following the implementation of the tell-show-do technique combined with non-verbal communication and play-based interaction. Establishing trust and gradual desensitization allowed the patient to feel safe and engage positively with the clinician. Patient identity has been protected to ensure privacy.

The tell-show-do technique was utilized using simple language, demonstration of instruments, and gradual behavioral adaptation, resulting in improved patient cooperation. Figure 6 depicts successful behavioral adaptation achieved through the use of structured communication and gradual desensitization techniques.

**Figure 7:**
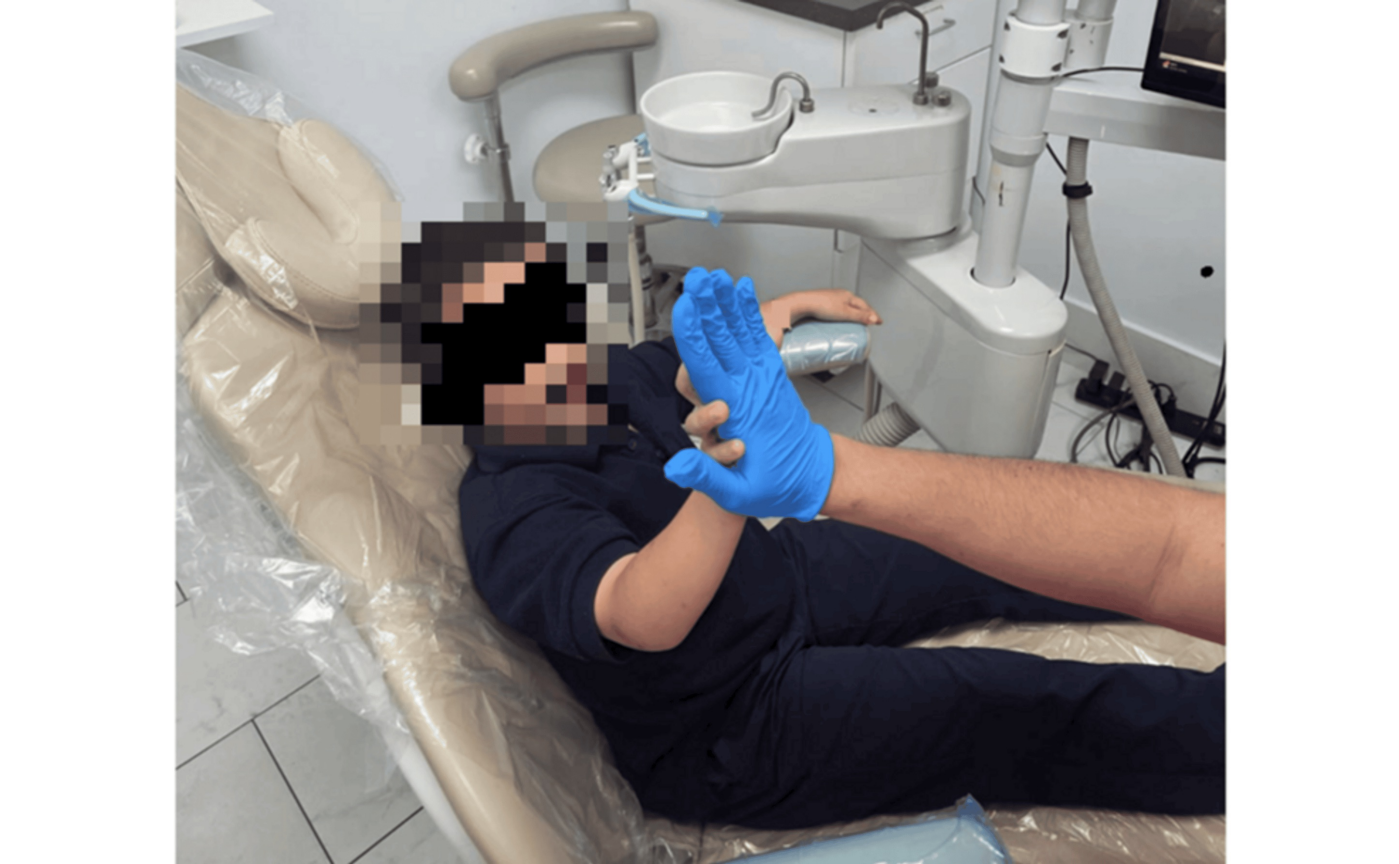
Maintenance of cooperation through consistent behavioral guidance in a child with autism spectrum disorder Continued use of non-verbal communication and the tell-show-do technique reinforced the child’s cooperation and trust. Consistency in clinician behavior and communication is essential to maintain a stable and predictable environment, allowing the patient to remain comfortable and engaged during dental care. Patient identity has been protected to ensure privacy.

Continued use of consistent behavioral guidance techniques reinforced patient trust and maintained cooperation throughout subsequent visits. Figure 7 presents the maintenance of patient cooperation through consistent behavioral management and supportive clinician interaction during dental treatment.

**Figure 8:**
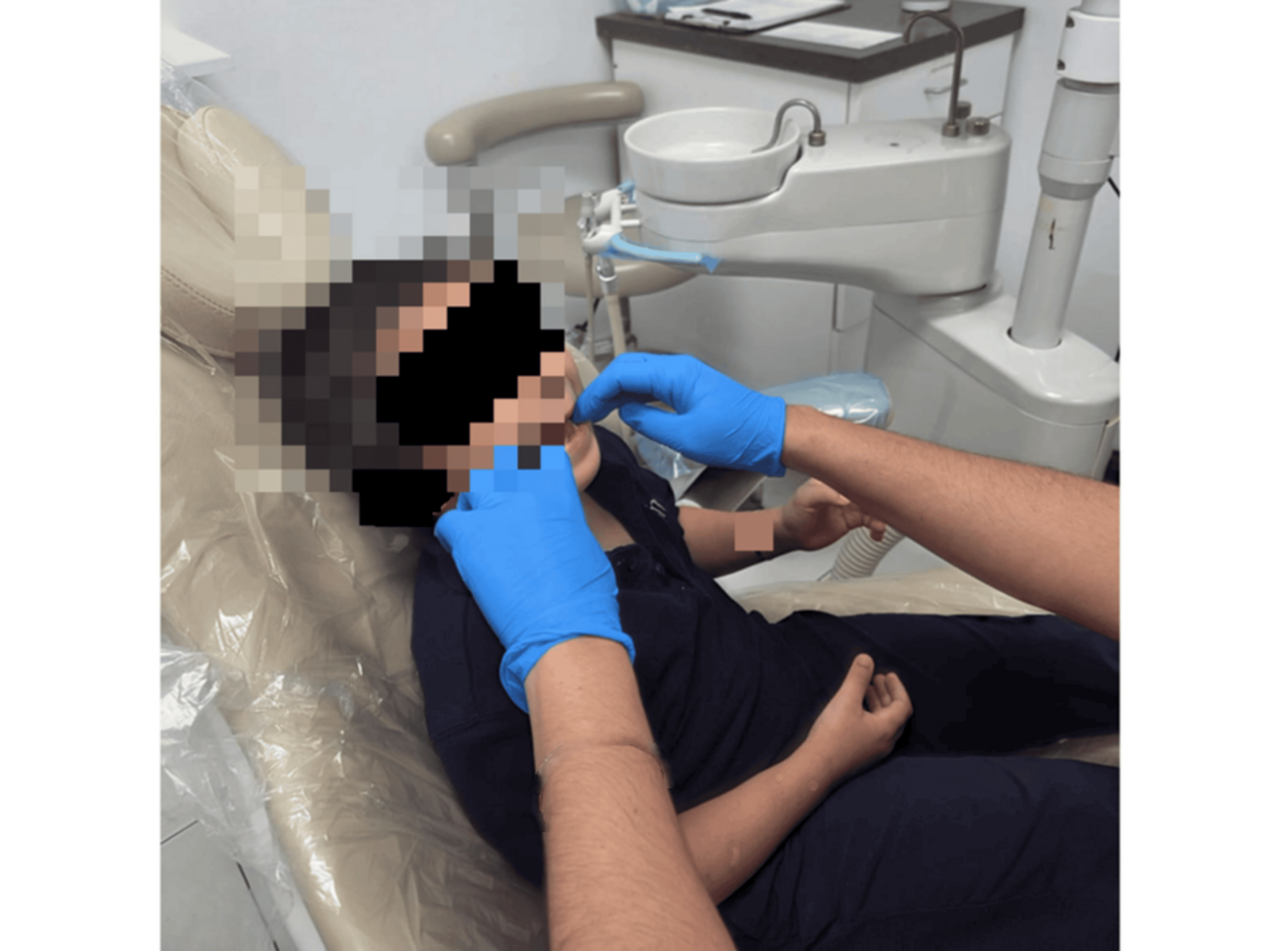
Successful acceptance of intraoral examination following behavioral management in a child with autism spectrum disorder Ultimately, the child achieved full cooperation, permitting intraoral examination and the commencement of dental treatment. The systematic application of behavioral management techniques, including desensitization, non-verbal communication, tactile guidance, and the tell-show-do approach, resulted in a significant reduction of anxiety and improved tolerance to intraoral procedures. This progression allowed the clinician to safely initiate both diagnostic and therapeutic interventions.

Over multiple visits, the child demonstrated progressive improvement in tolerance and cooperation. Preventive procedures, including prophylaxis and topical fluoride application, were successfully completed. Figure 8 demonstrates the successful initiation of intraoral dental treatment following implementation of individualized behavioral management techniques. Restorative treatment of carious lesions was subsequently performed with minimal resistance and without the need for sedation or general anesthesia.

**Figure 9:**
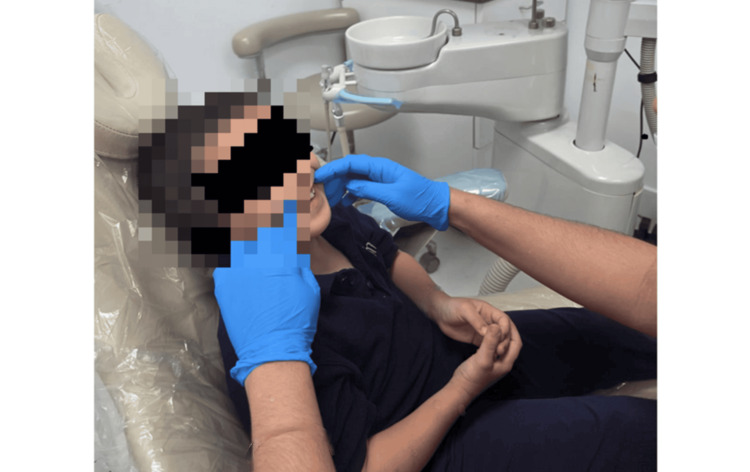
Continued cooperation during ongoing dental treatment in a child with autism spectrum disorder Ongoing dental treatment with satisfactory child cooperation. Consistent use of behavioral techniques and a stable clinician approach were maintained to ensure the child’s comfort and engagement. These techniques should be adapted to individual needs, as each child with autism spectrum disorder presents unique behavioral and sensory responses.

The child maintained cooperation during ongoing treatment, demonstrating the effectiveness of individualized behavioral management techniques to facilitate and sustain dental care. Figure 9 highlights continued child cooperation and adaptation throughout ongoing dental treatment within a structured and supportive clinical environment.

## Discussion

The management of children with ASD presents unique challenges in dental practice because of communication difficulties, behavioral responses, and reduced cooperation during clinical care [1,2]. Sensory sensitivities are also important barriers in the dental setting, as sounds, lights, and unfamiliar stimuli may increase anxiety and interfere with cooperation [3]. Environmental modifications and sensory-adapted dental settings have been shown to improve tolerance and reduce behavioral distress during dental treatment [4].

Children with ASD may present an increased risk of oral health problems, including dental caries and gingival inflammation, because of difficulties maintaining oral hygiene routines and reduced tolerance for professional dental care [5]. In this case, the child initially exhibited significant behavioral barriers, including refusal to sit in the dental chair and hypersensitivity to auditory stimuli. The implementation of a structured, individualized, non-pharmacological behavioral management approach resulted in progressive improvement in cooperation, ultimately allowing completion of both preventive and restorative procedures without the need for sedation or general anesthesia.

Gradual desensitization was essential in reducing anxiety, allowing the child to become familiar with the dental environment in a controlled and non-threatening manner [6]. The tell-show-do technique, caregiver involvement, predictable appointments, and positive reinforcement helped facilitate communication, improve understanding, and enhance trust during dental treatment [7].

Recent evidence further supports the effectiveness of structured behavioral guidance and dental adaptation strategies in improving cooperation and facilitating successful dental treatment in children with ASD [8]. Additional long-term evidence has highlighted the importance of individualized behavioral management approaches in improving oral health outcomes and treatment acceptance in children with ASD [9].

Sensory-adapted dental environments may further reduce anxiety and behavioral distress during clinical care [10]. Studies have also emphasized the importance of clinician preparedness and behavioral guidance techniques in improving treatment acceptance and reducing dental anxiety in children with ASD [11,12].

These findings reinforce the importance of individualized, child-centered approaches when managing children with special healthcare needs. While pharmacological interventions may be necessary in select cases, non-pharmacological techniques should be considered as a first-line approach because of their potential to promote long-term behavioral adaptation and positive dental experiences.

This case also emphasizes the critical role of consistency, routine, and behavioral guidance during clinical appointments. Early implementation of individualized behavioral management techniques may improve immediate treatment outcomes and long-term oral health by increasing acceptance of dental care and fostering positive attitudes toward future dental visits.

## Conclusions

The dental management of children with ASD can be successfully achieved through individualized, non-pharmacological behavior guidance and behavioral management techniques. This case report demonstrates that approaches such as desensitization, structured communication, the tell-show-do method, and caregiver involvement can significantly improve cooperation and facilitate completion of dental treatment without the need for sedation or general anesthesia. Establishing a consistent and supportive clinical environment plays a critical role in reducing anxiety and improving treatment outcomes. Early implementation of these behavioral strategies may contribute to improved long-term oral health and greater acceptance of dental care in children with special healthcare needs.

As a single-case report without standardized behavioral measurements, these findings should be interpreted cautiously and considered hypothesis-generating. Future studies incorporating objective behavioral outcomes, standardized behavioral assessment tools, and larger patient samples are recommended to further evaluate the effectiveness of behavioral management strategies in children with ASD.
